# Supplementary figures and images for: Changed Temporal Structure of Neuromuscular Control, Rather Than Changed Intersegment Coordination, Explains Altered Stabilographic Regularity after a Moderate Perturbation of the Postural Control System
Source: Entropy (Basel). 2019 Jun 21;21(6):614. doi: 10.3390/e21060614 (PMC7515107; doi:10.3390/e21060614)

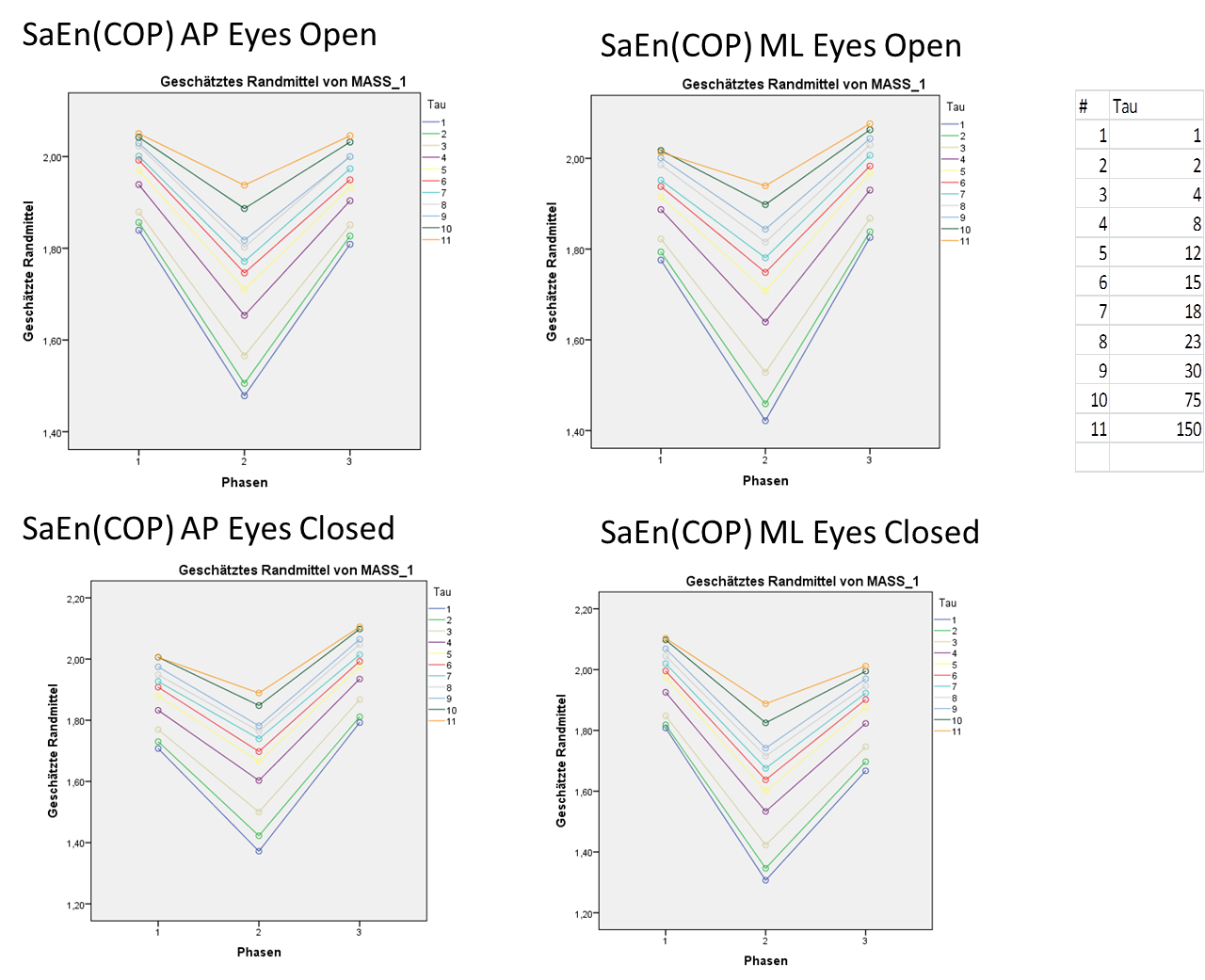

Supplement: Supplementary file 1 [file entropy-21-00614-s001.zip › Figure S1a.png]

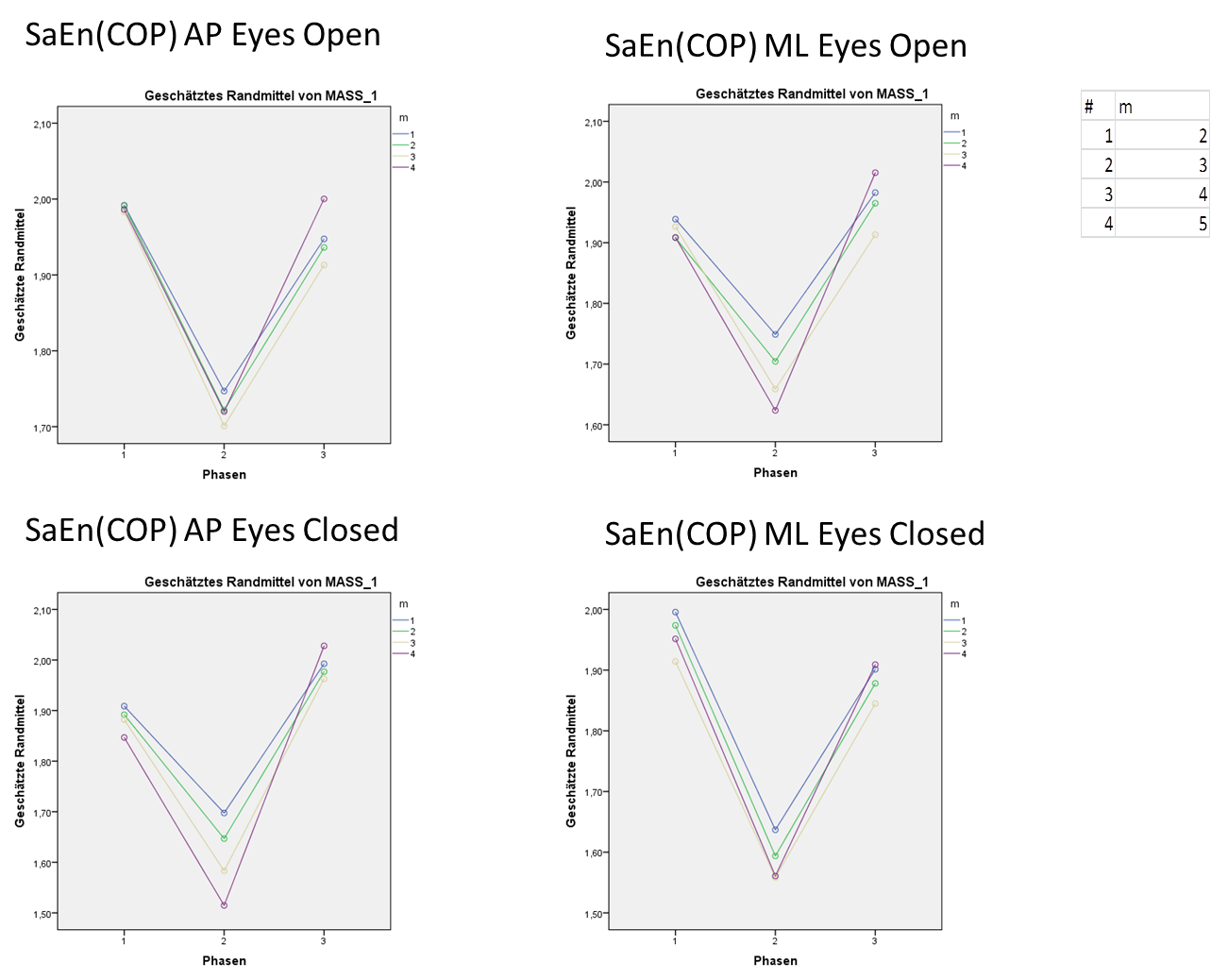

Supplement: Supplementary file 1 [file entropy-21-00614-s001.zip › Figure S1b.png]

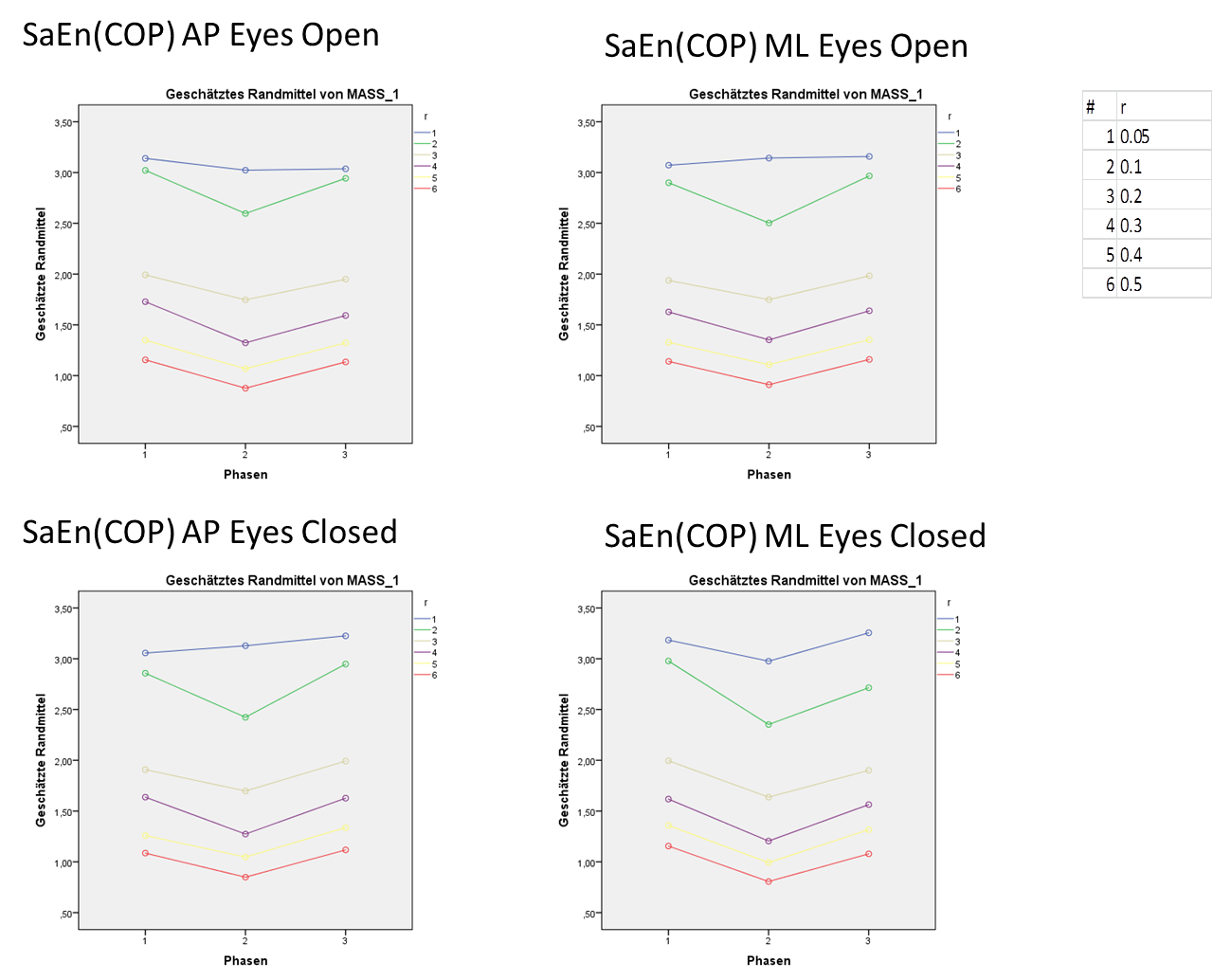

Supplement: Supplementary file 1 [file entropy-21-00614-s001.zip › Figure S1c.png]
